# Multi-Strain Probiotics Alleviate Food Allergy-Induced Neurobehavioral Abnormalities by Regulating Gut Microbiota and Metabolites

**DOI:** 10.3390/nu17121955

**Published:** 2025-06-08

**Authors:** Shouxun Hu, Luanluan Li, Chunyan Zhou, Yue Zhang, Xiaodan Yu

**Affiliations:** 1 Department of Developmental and Behavioral Pediatrics, Shanghai Children’s Medical Center, School of Medicine, Shanghai Jiao Tong University, Shanghai 200127, China; hushouxun@alumni.sjtu.edu.cn (S.H.); liluanluan880829@163.com (L.L.); 13127715748@163.com (Y.Z.); 2 Translational Medicine Institute, Shanghai Children’s Medical Center, School of Medicine, Shanghai Jiao Tong University, Shanghai 200127, China; zhouchunyan0318@163.com

**Keywords:** food allergy, probiotics, neurobehavior, gut microbiota, metabolomics, amino acids metabolism

## Abstract

**Background and aim:** Neurobehavioral changes associated with food allergies have been reported, but the therapeutic effects of probiotics have not been fully explored. Our study aimed to investigate the impact of multi-strain probiotics on neurobehavioral outcomes and to elucidate the underlying mechanism via the microbiota-gut-brain axis. **Methods:** C57BL/6J Male mice were randomly divided into the following three groups: (1) control group; (2) OVA-sensitized group; (3) OVA-sensitized group treated with multi-strain probiotics (OVA + P). Anaphylactic reactions and behavioral abnormalities were assessed by histological, immunological, and behavioral analyses. To further elucidate the underlying mechanisms, the prefrontal cortex was collected for microglial morphological analysis, while serum and fecal samples were obtained for untargeted metabolomic profiling and 16S rDNA-based gut microbiota analysis, respectively. **Results:** Multi-strain probiotics significantly alleviated anaphylactic reactions in OVA-sensitized mice, as evidenced by reduced serum IgE levels, decreased Th2 cytokines, and reduced epithelial damage. Meanwhile, neurobehavioral symptoms were alleviated, including anxiety-like and depression-like behaviors, repetitive behaviors, social avoidance, and impaired attention. Mechanistically, probiotics administration suppressed production of inflammatory cytokines (TNF-α, IL-1β and IL-6) and inhibited activation of M1 microglia in the prefrontal cortex, which might contribute to neuron recovery. Furthermore, multi-omics analysis revealed that amino acid metabolism restoration in OVA + P mice, particularly carboxylic acids and derivatives, which was remarkably correlated with alterations in gut microbiota and behaviors related to FA. **Conclusions:** Gut microbiota and its amino acid metabolites mediate the therapeutic effects of multi-strain probiotics on FA-induced behavioral abnormalities. These effects occur alongside the suppression of neuroinflammation and microglial activation in the prefrontal cortex. Our findings highlight the neuroimmune regulatory role of the gut-microbiota-brain axis and support the potential use of probiotics as an intervention for FA-induced brain dysfunctions.

## 1. Introduction

Food allergy (FA) is a pathological immune response to food-derived allergens [1] and a significant public health issue, affecting nearly 10% of children [2,3]. While FA is primarily known for its gastrointestinal, respiratory, and skin symptoms [4], emerging evidence links it to neurobehavioral disorders, such as anxiety-like and depression-like behaviors [5], attention deficit hyperactivity disorder (ADHD) [6], and autism spectrum disorder (ASD) [7]. Our earlier epidemiological work further supported this connection, showing infantile allergic diseases, including FA, were associated with an increased risk of delayed social-emotional development in early childhood [8]. However, the underlying mechanisms remain poorly understood.

In our previous work, we established an FA mice model with OVA (ovalbumin), one of the main allergens in eggs. Our experiments suggested that FA-induced behavioral abnormalities may involve the microbiota-gut-brain axis (MGBA), possibly through gut microbiota and amino acids metabolites (unpublished yet). Growing evidence supports the gut microbiome-FA connection, as individuals with FA exhibit distinct microbiome profiles compared to healthy controls. Microbial interventions—such as diet, probiotics, prebiotics, synbiotics, and fecal microbiota transplantation (FMT)—may help restore the gut microbiome structure [9]. Meanwhile, studies have established a link between neurobehavioral disorders and gut microbiota. For example, fecal microbiota from ASD patients could induce autism-like behaviors in mice, disrupt amino acids metabolism and alter ASD-relevant gene expression in the brain. Treatment with specific microbial-derived amino acid metabolites has been shown to improve behavioral deficits in mouse models [10].

These findings indicate that microbiota-targeted interventions could alleviate not only allergic symptoms but also FA-related neurobehavioral impairments. Probiotics are living microorganisms that confer healthy benefits when administered in adequate amounts; they might offer a cost-effective approach to restore microbial dysbiosis. For example, common probiotic strains like *Bifidobacterium* and *Lactobacillus* have therapeutic potential in food allergies by restoring the intestinal epithelial barrier and regulating immune cells [11,12]. Several studies also found that probiotics may improve neurobehavioral symptoms through neurotransmitter modulation and neuroinflammatory control [13,14]. However, the interplay between probiotic supplementation, FA, and neurobehavioral outcomes remains unclear. Moreover, limited studies have employed multi-omics approaches to investigate the MGBA changes in FA models.

Therefore, we investigate whether multi-strain probiotics could alleviate FA-induced behavioral abnormalities in a mouse model. We comprehensively assessed the following: allergic responses and behavioral changes; systemic cytokine levels and neuroinflammation; gut microbial composition (via 16s rDNA sequencing of the V3–V4 region); serum metabolomics alterations (focusing on amino acid metabolism). Our results provide novel insights by which the MGBA regulates neuroimmune response in FA, supporting probiotics as a promising therapeutic approach for FA-related brain dysfunctions.

## 2. Materials and Methods

### 2.1. Animals

Three- to four-week-old C57BL/6J male mice were purchased from Shanghai Jihui Laboratory Animal Technology Co., Ltd. (Shanghai, China) (certificate number: SCXK 2022-0009). Mice were randomly divided into OVA (ovalbumin induced food allergy) group, control group and OVA + P (ovalbumin induced food allergy + multi-strain probiotics administration) group, housed under a 12 h light/12 h dark cycle in a specific-pathogen-free environment, and fed by OVA-free food and water ad libitum.

### 2.2. Experimental Protocol

**Figure 1:**
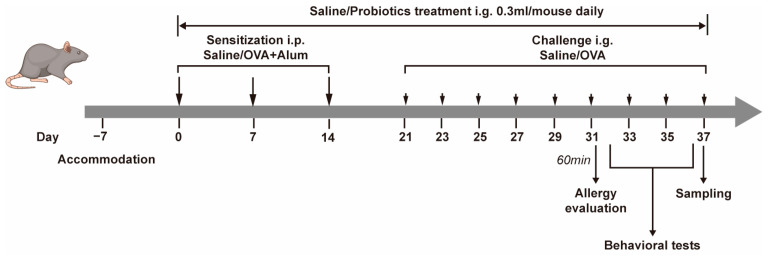
Schematic timeline of experimental procedure.

The experiment protocol is displayed in Figure 1. Briefly, OVA mice and OVA + P mice were sensitized with 100 μg ovalbumin (OVA grade V, Sigma-Aldrich, St. Louis, MO, USA, A5505) and 2 mg aluminum adjuvant (Sigma-Aldrich, St. Louis, MO, USA, 239186) via intraperitoneal injection (i.p.) on day 0, 7, and 14, and then the mice were challenged every other day with 60 mg OVA by intragastric gavage (i.g.) from day 21 to day 39. Control mice were only sham sensitized with 2 mg aluminum adjuvant by i.p. and challenged with PBS. While the mice assigned to OVA + P group received additional treatment, a daily oral dose of multi-strain probiotics (10^9^ CFU/300 μL/mouse/day) from day 0 to 37. The mixture of probiotics contained five live, freeze-dried bacterial strains: *Bifidobacterium animalis* Bb-12, *Bifidobacterium lactis* HN019, *Bifidobacterium breve* M-16V, *Lactobacillus rhamnosus* HN001, *Lactobacillus acidophilus* NCFM. At the end of treatment, mice were subjected to behavioral tests and then sacrificed for tissue and blood collection. The body weights were recorded throughout the experiment, and physical symptoms were observed and scored according to the rules listed in Table S1 within 60 min after the challenges. Rectal temperature was also obtained after challenge.

More details are shown in the Supplementary Methods.

## 3. Results

### 3.1. Multi-Strain Probiotics Attenuated Allergic Responses in OVA Mice

**Figure 2:**
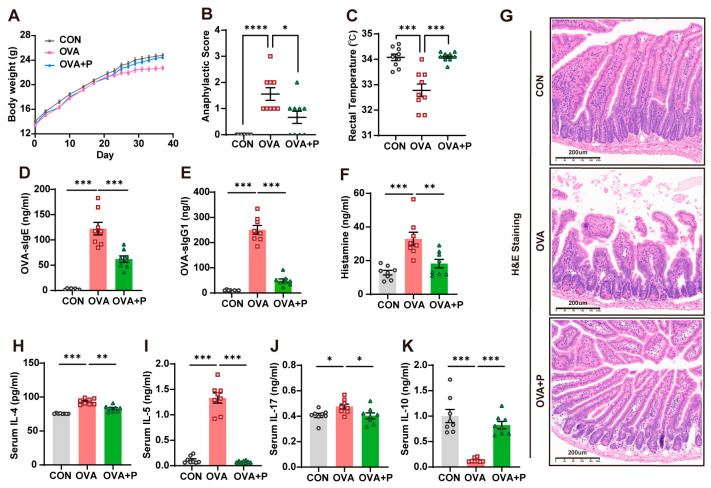
Multi-strain probiotics attenuated allergic responses in OVA mice. (**A**) The gains of body weights. (**B**) Anaphylactic symptoms. (**C**) Rectal temperature. (**D**) Serum OVA-specific IgE. (**E**) Serum OVA-specific IgG1. (**F**) Serum histamine. (**G**) Representative images of intestinal morphology by H&E staining. (**H**) Serum IL-4. (**I**) Serum IL-5. (**J**) Serum IL-17. (**K**) Serum IL-10. *n* = 8–9 in each group. Data are presented as the means ± SEM. * *p* < 0.05, ** *p* < 0.01, *** *p* < 0.001 and **** *p* < 0.0001 using Mann-Whitney U test.

At first, we evaluated the protective effect of multi-strain probiotics in FA in OVA mice (Figure 2). After the OVA challenge, allergic mice exhibited reduced body weight gain compared to controls. Compared to the OVA mice, multi-strain probiotics significantly restored growth (Figure 2A). OVA mice exhibited higher anaphylactic scores and lower core body temperature than control mice. Probiotic treatment significantly alleviated anaphylactic responses, as evidenced by lower anaphylactic scores and restored core body temperature. (Figure 2B,C).

Allergen-sIgE is the main indicator for the clinical diagnosis of FA. To determine whether multi-strain probiotics could suppress the development of FA, we measured OVA-sIgE, OVA-sIgG1 and histamine levels in serum. Mice in the OVA group produced significantly higher levels of OVA-IgE, OVA-IgG1 and histamine in serum than mice in control group; all of these were lowered by probiotic treatment (Figure 2D–F).

H&E staining of intestinal sections revealed that morphological damages occurred in the OVA group, including mucosal atrophy and edema, epithelial detachment and villous atrophy. As expected, multi-strain probiotics led to significant improvement in the disruption of intestinal structure (Figure 2G).

In this study, we found that OVA + P mice displayed lower immune allergic responses than the OVA group. According to the ELISAs results, multi-strain probiotics could significantly suppress aberrant Th2- and Th17-type responses via inhibition of IL-4, IL-5 and IL-17 secretion (Figure 2H–J). In contrast, multi-strain probiotics treatment significantly increased IL-10 levels compared to the OVA group (Figure 2K). Our results indicated that multi-strain probiotics could affect the immune response of OVA mice by shifting a polarized Th2/Th17 response to a Treg-type profile.

### 3.2. Multi-Strain Probiotics Partially Mitigated Neurobehavioral Abnormalities in OVA-Induced FA

**Figure 3:**
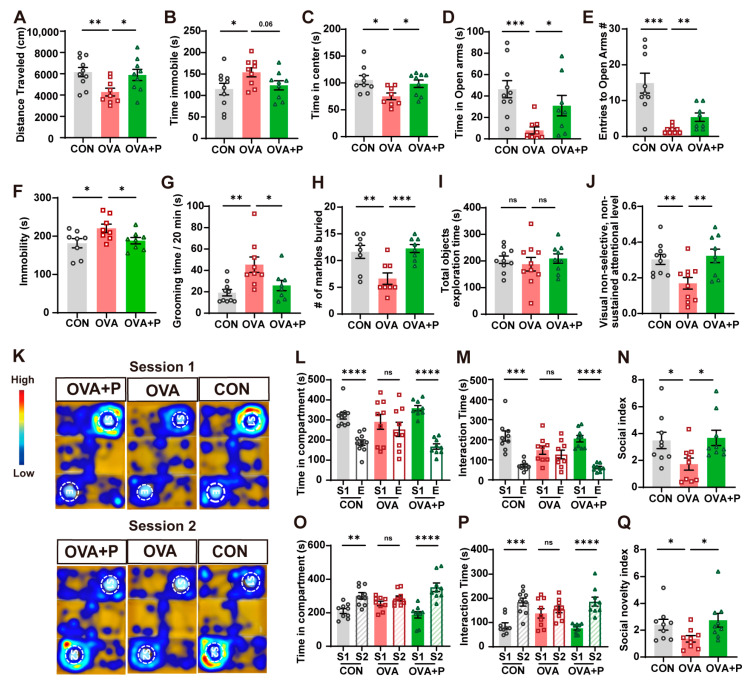
Positive effect of multi-strain probiotics in neurobehaviors. (**A**–**C**) Open Field Test (OFT). (**D**,**E**) Elevated plus maze (EPM). (**F**) Forced swimming test (FST). (**G**) Self-grooming. (**H**) Marble burying test. (**I**,**J**) Non-sustained, non-sustained visual attention test (NNAT). The NNAT attention level = [(time spent exploring new object)/(total time spent exploring objects)] × 100%. (**K**) Representative trajectory heatmap of mice in session 1 and session 2 of three-chamber sociability test (TST). S1: Stranger mice 1. S2: Stranger mice 2. E: Empty cup. (**L**–**N**) TST in Session 1. Social index (SI) = [(time spent interaction with stranger 1)/(time spent interaction with empty cup)] × 100%. (**O**–**Q**) TST in Session 2. Social novelty index (SNI) = [(time spent interaction with stranger 2)/(time spent interaction with stranger 1)] × 100%. *n* = 8–12 in each group. Data are presented as the means ± SEM. ns = not significant. * *p* < 0.05, ** *p* < 0.01, *** *p* < 0.001 and **** *p* < 0.0001 using Mann-Whitney U test.

Our previous work identified the neurobehavioral alterations in FA mice. Here, we evaluated the protective effect of multi-strain probiotics on neurobehavioral outcomes via a series of behavior tests. After the challenge, control and OVA + P groups showed greater locomotor activity, traveling longer distances and spending less time immobile in OFT compared to OVA mice (Figure 3A,B). Conversely, OVA mice showed more anxiety and spent less time in the central area of the OFT (Figure 3C). Consistent with the OFT, OVA mice exhibited reduced exploration in EPM, spending less time in the open arms and making fewer open-arm entries (Figure 3D,E), along with increased depressive-like behaviors as evidenced by prolonged immobility in the FST (Figure 3F). Importantly, probiotics treatment significantly improved these behaviors, increasing center time in the OFT, open-arm exploration in the EPM, and reducing immobility in the FST, indicating effective mitigation of anxiety- and depressive-like behaviors in FA mice.

We next examined the effect of probiotics on repetitive self-grooming behavior in FA mice. The OVA group displayed significantly longer self-grooming durations compared to controls, and treatment of multi-strain probiotics could partially mitigate this behavioral deficit (Figure 3G). Consistent with previous reports of reduced digging activity in FA models [15], we also observed fewer buried marbles in OVA mice compared to controls. Notably, treatment of multi-strain probiotics improved digging behavior (Figure 3H).

The non-selective, non-sustained visual attention test (NNAT) was performed to evaluate attentional performance. Following familiarizations, all three groups showed comparable total object exploration times (Figure 3I). However, OVA mice exhibited significantly impaired visual non-selective, non-sustained attentional levels compared to controls. Importantly, multi-strain probiotics treatment significantly improved attentional level (Figure 3J).

Finally, alterations in social behavior and communication were investigated via a three-chambered test. In the social ability test (session 1), both control and OVA + P mice showed an obvious preference for interacting with stranger mice 1, whereas OVA mice showed reduced social interest, as evidenced by their lower social index (Figure 3L,M). Consistently, in the social novelty preference test (session 2), control and OVA + P mice spent substantially more time interacting with the novel stranger mice 2 compared to OVA mice (Figure 3O,P). Our data indicated that probiotic treatment partially rescued both sociability and social novelty preference in FA mice. (Figure 3N,Q).

### 3.3. Multi-Strain Probiotics Attenuated FA-Induced Neuroinflammation and Neuronal Injury

**Figure 4:**
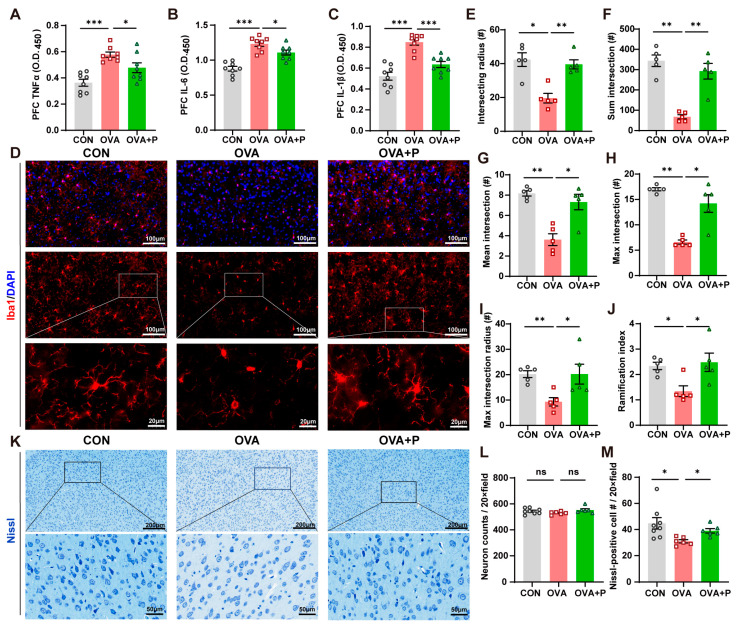
Multi-strain probiotics alleviated neuroinflammation and neural damage in prefrontal cortex. (**A**) TNF-α in PFC. (**B**) IL-6 in PFC. (**C**) IL-1β in PFC. (**D**) Representative images displaying the morphological changes in microglia in PFC. (**E**–**J**) Sholl analysis: (**E**) intersecting radii, (**F**) sum intersection, (**G**) mean intersection, (**H**) max intersection, (**I**) max intersection radius and (**J**) ramification index. *n* = 5 in each group. (**K**) Representative images displaying the neuron changes in Nissl stain. (**L**) Neuron counts in PFC. (**M**) Nissl-positive cell counts per 20× field. *n* = 5 in each group. Data are presented as the means ± SEM. ns = not significant. * *p* < 0.05, ** *p* < 0.01, *** *p* < 0.00 using Mann-Whitney U test.

As the prefrontal cortex (PFC) is an important region involved in memory, mood, and behavior regulation [16], we further detected neuroinflammation and neuronal damage in this area to explore the possible causes of behavioral abnormalities. First, the levels of pro-inflammatory cytokines, i.e., TNF-α, IL-6 and IL-1β, were remarkably increased in OVA mice compared to that in control mice, whereas multi-stain probiotics treatment significantly decreased these cytokines (Figure 4A–C). As brain macrophages regulate brain development, the maintenance of neuronal networks, and injury repair [17], the number of microglia in the PFC did not differ significantly among the three groups (Supplementary Figure S1A). Then, we conducted Sholl analysis on the Iba1-positive cells to distinguish morphological changes and microglial activation in three groups. We observed that, in the PFC of the OVA mice, microglia cells showed a reduced morphological complexity, suggesting an activated, pro-inflammatory phenotype (M1), compared to the control and OVA + P groups (Figure 4D). In more detail, we detected the microglial activation in OVA mice via following morphological changes: decreased intersecting radius, sum intersection, mean intersection, max intersection, max intersection radius, ramification index and ending radius, and increased area soma (Figure 4E–J, Supplementary Figure S1B,C). These indicated that multi-strain probiotics administration might associate with suppression of microglia in PFC. We further identified neuronal injury after the OVA challenge (Figure 4K). Although no difference was found in total neuron counts among the three groups (Figure 4L), Nissl bodies in PFC were decreased in the OVA mice compared to controls, indicating neuron damage in the PFC induced by FA. Moreover, Nissl bodies in the OVA + P group were protected, possibly because of multi-strain probiotics (Figure 4M).

### 3.4. Multi-Strain Probiotics Partially Restored the Structure of Gut Microbiota

**Figure 5:**
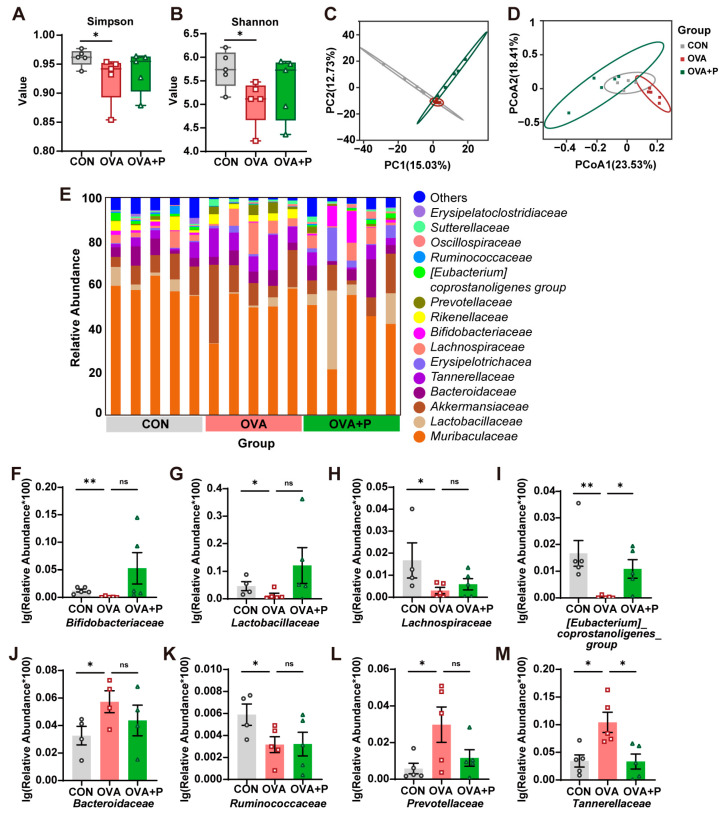
Imbalance of gut microbiota in OVA-induced FA mice. (**A**,**B**) Alpha diversity analysis: (**A**) Simpson and (**B**) Shannon. (**C**,**D**) Beta diversity analysis performed by (**C**) PCA and (**D**) PCoA. (**E**) Different gut bacterial composition at family level in three groups. (**F**–**M**) The relative abundance of 8 bacteria in three groups at family level. *n* = 5 in each group. Data are presented as the means ± SEM. ns = not significant. * *p* < 0.05, ** *p* < 0.01.

To explore the effects of multi-strain probiotics administration on the gut microbiota of mice, 16s rDNA amplicon sequencing was performed. The alpha diversity (Simpson, Shannon) was analyzed (Figure 5A,B). The OVA mice caused a decrease in the gut microbiota diversity in mice, and multi-strain probiotics partially restored the richness. Meanwhile, we used beta diversity to measure gut microbiota differences between groups. Multivariate cluster analysis based on bacterial OTUs levels implied that OVA-induced FA affected the composition of the gut flora in mice. There were some differences between the control/OVA, OVA + P/OVA, but the differences were not significant (Figure 5C). PCoA was performed according to Bray–Curtis dissimilarity and calculated based on the abundance of OTUs, which showed significant disparities among the intestinal flora of the three groups (PERMANOVA: CON vs. OVA, R^2^ = 0.293, P = 0.013; OVA vs. OVA + P, R^2^ = 0.295, P = 0.009; CON vs. OVA + P, R^2^ = 0.216, P = 0.011) (Figure 5D).

To investigate the association between OTUs and OVA allergy in mice, we analyzed the compositional distribution of the intestinal flora in the three groups at the family level (Figure 5E) and screened 16 differential OTUs with relative abundance >0.1%. Compared to the control group, our data showed that the OVA group downregulated five bacterial proportions at the family level, including *Bifidobacteriaceae*, *Lactobacillaceae*, *Lachnospiraceae*, *[Eubacterium]_coprostanoligenes_group* and *Ruminococcaceae*, and upregulated three bacterial proportions, including *Bacteroidaceae*, *Prevotellaceae* and *Tannerellaceae*. However, the supplementation of multi-strain probiotics only partially restored these changes. We reported uncorrected results in Figure 5F–M, because FDR-corrected results were non-significant (Table S2). Together, these data show that multi-strain probiotics could partially restore the structure of gut microbiota.

### 3.5. Multi-Strain Probiotics Altered Amino Acid Metabolism in OVA Mice

**Figure 6:**
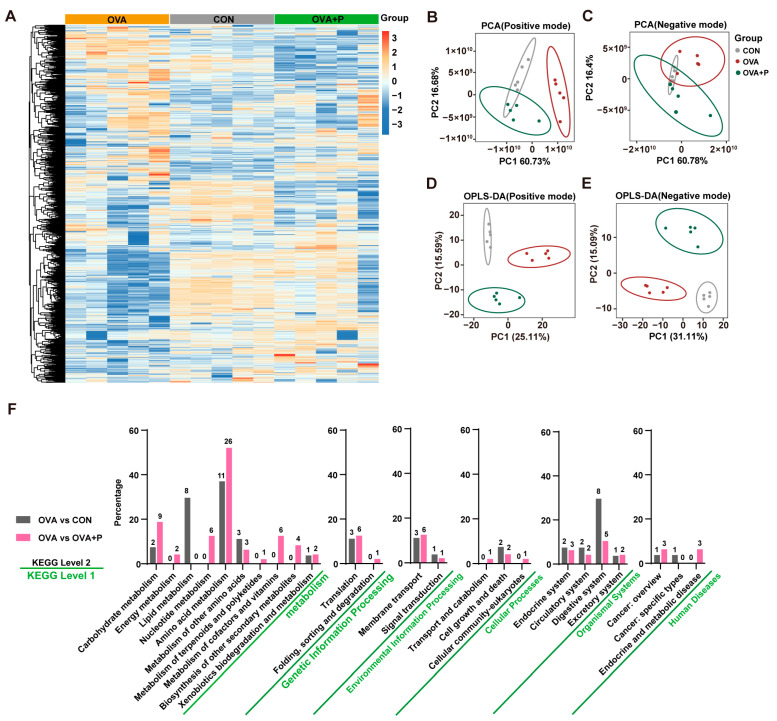
Effects of OVA-induced FA on metabolic differences in the serum. (**A**) The heatmap of serum metabolites level in three groups. (**B**–**E**) Unsupervised PCA was performed in serum metabolites of three groups in (**B**) positive mode and (**C**) negative mode. Supervised PCA analysis (OPLS-DA) showed differences in serum metabolism groups within three groups in (**D**) positive mode and (**E**) negative mode. (**F**) The involved pathway enriched by significantly differential metabolites in KEGG level 1 and level 2.

In global metabolomic profiling, a total of 1291 metabolites were identified and quantified by GC-TOF/MS analysis (Figure 6A). PCA showed inconsistencies in the serum metabolic profiles among three groups, and the score plots are shown in Figure 6B,C. We further calculated the OPLS-DA model and showed significant clustering between the control/OVA groups, OVA/OVA + P group (Figure 6D,E), with good ability to distinguish between the different serum samples (Positive mode: control vs. OVA, R^2^Y = 1.00, Q^2^Y = 0.85; OVA vs. OVA + P, R^2^Y = 1.00, Q^2^Y = 0.68; control vs. OVA + P, R^2^Y = 0.99, Q^2^Y = 0.80; Negative mode: control vs. OVA, R^2^Y = 1.00, Q^2^Y = 0.85; OVA vs. OVA + P, R^2^Y = 1.00, Q^2^Y = 0.68; control vs. OVA + P, R^2^Y = 0.99, Q^2^Y = 0.82).

To explore the key metabolism pathway, we compared the metabolites results with the KEGG database. Figure 6F showed that 24 pathways of level 2 were altered and mainly concentrated in metabolism (level 1). The percentage for each level 2 pathway showed that major altered metabolites (*p* < 0.05) enriched in amino acid metabolism. Compared to the OVA group, the control group and OVA + P group screened out 11 and 26 differential metabolites belong to amino acid metabolism, respectively.

### 3.6. Correlation Between Altered Serum Amino Acid Metabolites and Gut Microbiota

**Figure 7:**
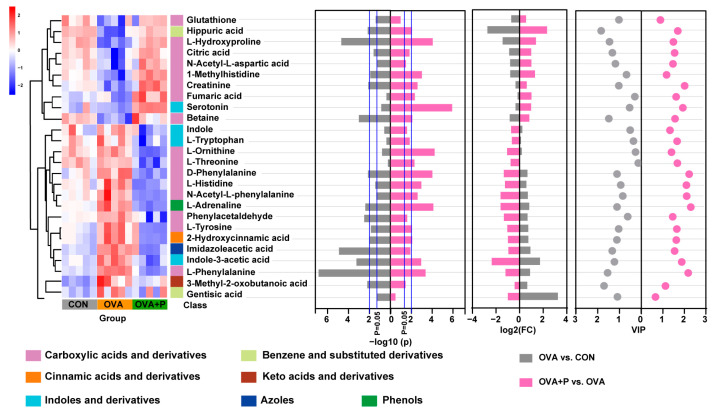
Heatmap obtained by Pearson’s hierarchical clustering of metabolites selected from carbohydrate metabolism and amino acid metabolism based on *p* value < 0.05.

**Figure 8:**
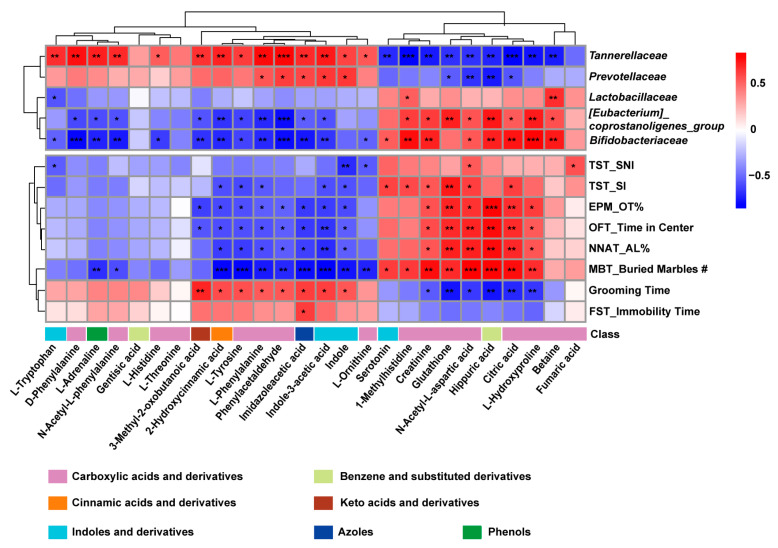
Correlation analysis between different gut microbiota and serum metabolites with behavioral index. * *p* < 0.05, ** *p* < 0.01, *** *p* < 0.001.

The 26 differential metabolites in amino acid metabolism among the three groups were further selected for hierarchical Pearson’s clustering. Consistent with the results of PCA and OPLS-DA, hierarchical Pearson’s clustering analysis also showed significant disparities between the control/OVA groups or OVA/OVA + P groups. We found that most of the altered metabolites were carboxylic acids and derivatives (Figure 7). Some of those metabolites, including phenylalanine, glutathione, N-acetyl-L-aspartic acid (NAA) and L-Hydroxyproline, have been proven to play important roles in immune homeostasis and neuron function. To explore the possible link between gut microbiota, amino acid metabolism, and behavioral outcomes, Pearson’s correlation between differential metabolites in the serum and differential OTUs at the family level, behavioral index were analyzed (Figure 8). A highly significant correlation was presented between carboxylic acids and derivatives and the five differential OTUs, particularly *Bafidobacteraceae*, *[Eubacterium]_coprostanoligenes_group* and *Tannerellaceae*. As for behavioral index, we observed significant correlations between carboxylic acids and derivatives and social behaviors (SI, SNI), anxious-like behaviors (OT% of EPM, time in center of OFT), attention level (NNAT), digging behaviors (MBT) and compulsive repetitive behaviors (grooming time).

## 4. Discussion

This study provides evidence that multi-strain probiotics can mitigate both physiological and psychological manifestations of FA in a mouse model. Specifically, probiotic supplementation was able to ameliorate allergic responses, gastrointestinal symptoms, anxiety- and depressive-like behaviors, stereotyped behavior, social avoidance, and attention deficiency. We further revealed that multi-strain probiotic supplementation partially restored FA-induced gut dysbiosis and altered amino acid metabolism. In addition, probiotic supplementation exerted anti-inflammatory and neuroprotective effects, potentially mediated through the modulation of cytokine signaling and suppression of microglial activation in the prefrontal cortex (Supplementary Figure S2).

Previous human and animal studies have reported the positive effects of probiotic supplementation in psychological disorders such as depression, anxiety [18], and autism [13]. For example, Schaub et al. applied an 8-strain probiotic mixture on depressed patients, including *Bifidobacterium breve*, *B. longum*, *B. infantis* and *Lactobacillus acidophilus*. They found probiotics increased the abundance of the genus *Lactobacillus* in the intervention group, which was associated with decreased depressive symptoms and putamen activation [19]. After administration with a mixture of four probiotic strains including *B. infantis* Bi-26, *B. lactis* BL-04, *L. rhamnosus* HN001, and *L. paracasei* LPC-37, children with autism could also ameliorate ASD symptoms. This therapeutic effect was achieved via suppression of pathogenic bacteria, stimulation of short-chain fat acids production and modulation of hyperserotonergic state and dopamine metabolism disorder [13]. The positive effects of probiotics have also been proved in animal models with emotional and behavioral abnormalities. Probiotics strains from *Bifidobacterium* and *Lactobacillus* have demonstrated positive effect in alleviating anxiety or depression symptoms across diverse etiologies, including postnatal maternal separation [14], ethanol exposure [20,21], chronic stress [22], chronic sleep disruption [23], menopause [24], inflammatory bowel disease [25]. Similarly, those two genus probiotics could also alleviate autistic-like behavioral phenotypes caused by early-life stress [26], valproic acid exposure [27,28], and genetic abnormality [29,30]. In line with those studies, we first evaluated and showed the positive modulation effect of probiotics on neurobehavioral abnormalities in FA mice model. Notably, there are some limited clinical trials suggesting that administration of probiotics in early life may exert long-term protection on preventing allergic diseases, such as atopic dermatitis [31]. However, the research about long-term maintenance of neurobehavioral improvement induced by probiotics is scarce. Nearly all of the human and animal studies focused on the short-term or immediate effect of probiotics; thus, it would be useful to investigate its prolonged effect, particularly on neurobehaviors.

Growing evidence supports bidirectional gut–brain axis communication, where gastrointestinal dysfunction and neurobehavioral disorders mutually influence each other. This mechanistic interplay suggests the gut might be a shared target for neuropsychiatric disorders. Specifically, children with ASD had a greater risk of gastrointestinal symptoms, including abdominal pain, gaseousness/bloating, constipation, diarrhea, vomiting, and food allergy/intolerance [32,33]. Patients with anxiety and depression were more likely to have gastrointestinal dysfunction with impaired intestinal barrier [34,35,36]. Both depressed symptoms and gastrointestinal functions were improved after intervention with multi-probiotics, including *Bifidobacterium breve* CCFM1025, *Bifidobacterium longum* CCFM687, and *Pediococcus acidilactici* CCFM6432 [22]. In murine models of ASD and anxiety/depression, they also found increased intestinal permeability, elevated neutrophil infiltration and pro-inflammatory cytokines also exhibited [37,38,39]. It has also been proved in colitis mice model and genetic mice model that *Bifidobacterium* and/or *Lactobacillus* have the ability to alleviate the colitis and neuropsychiatry at the same time [30,39,40]. Li et al. further demonstrated that congenitally underdeveloped intestine was an early driving force shaping the ASD-like phenotype. Attenuation of intestinal oxidative stress led to the amelioration of autism-related behaviors [41]. In our study, gastrointestinal symptoms were assessed with neurobehaviors, including anaphylactic symptoms and intestinal pathology. Similarly, after administration with a mixture of *Bifidobacterium* and *Lactobacillus*, improved mood, ASD-like behavior, and attention were also detected, along with improved FA clinical manifestations and intestinal morphology. Our results reinforce the strong association between neurobehavioral abnormalities and gastrointestinal distress. Specifically, we demonstrate that *Bifidobacterium* and *Lactobacillus*, show particular promise for simultaneously improving both neurological and gastrointestinal outcomes. We provide evidence that the gut represents a novel, shared therapeutic target for managing diverse neuropsychiatric disorders, with probiotic interventions offering a particularly cost-effective approach.

Alterations of the intestinal microbiota have been corelated with gastrointestinal distress and neurobehavioral disorders. Our study demonstrated that the supplementation of *Bifidobacterium* and *Lactobacillus* exerted a beneficial effect on gastrointestinal manifestations, neurobehavioral abnormalities, and inflammatory priming in FA mice. To this regard, we observed that the gut microbiota structure (α diversity), which was significantly different between control and allergic mice, was partially restored to a physiological level after multi-strain probiotics treatment. This suggested that the positive effects of probiotics may be mediated through re-modulation of the intestinal microbiota. Multi-strain probiotics supplementation decreases the abundance of *Bacteroidaceae*, *Prevotellaceae* and *Tannerellaceae*, which are considered harmful. Indeed, elevated abundance of *Bacteroidaceae* was correlated with intestinal ferroptosis and Th2-type systemic inflammation [42]. *Prevotellaceae*’s impact on the gut is still controversial, but it was suggested that it could induce colonic inflammation by modulating the composition of secondary bile acids in the stool [43] and connect to the brain’s reward center [44]. *Tannerellaceae*, which secretes endotoxins in the gut, is positively correlated with pro-inflammatory cytokine TNF-α [45,46]. Our data showed a partially re-colonization in the gut of FA mice, in *Bifidobacteriaceae*, *Lactobacillaceae*, *Lachnospiraceae*, *Ruminococcaceae* and *[Eubacterium]_coprostanoligenes_group*, which are known to alleviate the inflammatory process. *Bifidobacteriaceae* and *Lactobacillaceae*, the regulators of amino acid metabolism [47,48,49], effectively alleviated gastrointestinal symptoms by mitigating intestinal epithelial cell damage, reduced allergic inflammation by changing the Th2/Th1 balance toward a dampened Th2 response [50,51,52]. *Lachnospiraceae* [53,54,55] and *[Eubacterium]_coprostanoligenes_group* [56,57], the SCFA-producing bacteria, perform a crucial role in gastrointestinal health, anti-inflammatory effects, and the development of allergic diseases. The association between *Ruminococcaceae* and indole metabolites is also one of the key factors in maintaining the integrity of the intestinal barrier and regulating the imbalance of Th2/Th1 proportion [58,59,60]. According to a previous study, all of the amino acid metabolism, indole metabolism and SCFA profoundly influence neurobehaviors and brain function [10,61,62].

The intestinal microbiota regulates activation and function of microglia [63]. In this study, a very important role of amino acid metabolites has been highlighted, as energy and metabolic intermediates, in the modulation of microglia in food allergic mice. The role of probiotics, particularly *Bifidobacterium* and *Lactobacillus*, in modulating the amino acid metabolism is well established [47,48,49]. Phenylalanine, glutathione, N-acetyl-L-aspartic acid (NAA) and L-Hydroxyproline belong to the carboxylic acids and derivatives. Our study found peripheral accumulation of phenylalanine, which has been reported to stimulate the differentiation and proliferation of pro-inflammatory T helper 1 (Th1) cells. The infiltrating peripheral Th1 cells may locally crosstalk with M1 microglial cells in the brain, resulting in pathological neuroinflammation and behavioral abnormalities [64]. The main probiotic strains of *bifidobacteria* and *lactobacilli* are capable of alleviating oxidative stress via metabolites [65]. The present study detected increased glutathione and citric acids in the probiotics treatment group. Glutathione plays a crucial role in the antioxidant defense system and the maintenance of redox homeostasis in brain, which are also significant for microglia activation and neurobehavior [66]. N-acetyl-L-aspartic acid (NAA) are major constituents of vertebrate brain, which has been reported to reduce symptoms in depressed patients [67] and patients with obsessive–compulsive disorder [68]. Although there is no research claiming the association between N-acetyl-L-aspartic acid and microglia, it may regulate differentiation of oligodendrocyte progenitor cells by stimulating histone deacetylase (HDAC) activity [69]. In addition, amino acids could promote cell proliferation and junction protein expression related to intestinal epithelium, thus accelerating barrier integrity restoration [70]. For example, Hydroxyproline, as a structurally and physiologically important amino acid in animals, plays an important role in cell antioxidative reactions, survival, and homeostasis [71]. Supplementation with Hydroxyproline could attenuate dextran sulfate sodium-induced colitis in mice by inhibiting the NF-кB/IL-6 [72]. According to a previous study, indole-3-acetic acid (IAA), derived from bacterial metabolism of tryptophan, was supposed to reduce microglial cytokine and chemokine production. Interestingly, we found that the concentration of IAA in serum was significantly elevated in FA mice, and restored after probiotic treatment, which differed from previous results. This discrepancy suggested that IAA might play a different role in FA pathogenesis and the underlying mechanism remains unresolved.

There are some limitations in this study. Firstly, we did not consider the sex difference. Our male-only finding might limit the generalizability, which needs further sex-specific experiments to validate. Secondly, the lack of FDR-significant taxa indicated that larger studies are needed to confirm these preliminary observations. Thirdly, we did not employ the FMT or gnotobiotic mouse models in this study to further prove the causality between specific microbial taxa and behavioral changes. Finally, we chose multi-strain probiotics because most clinical products available are multi-strain formulations. However, multi-strain probiotics ignore the strain-specific effect on allergic inflammation and neurobehavioral outcomes. Clinical application of probiotics in this field would face several challenges without clarifying the strain-specific effect. While the multi-strain probiotics used here likely worked together in reducing allergic inflammation and improving neurobehavioral outcomes, the strain-specific effects and probiotics interactions are also vital for product development. Additionally, probiotics face stringent regulatory requirements when converting from animal to human, particularly to children. Sufficient quality and long-term safety assessments for these “live biotherapeutic products” should be taken into consideration before expanding use into vulnerable populations. Moreover, animal behavioral tests were used widely and mimicked certain human behaviors, but they cannot fully replicate the heterogeneity and subjective experience of human disorders. Future studies should integrate objective biomarkers with standardized clinical scales to enhance translational reliability.

## 5. Conclusions

Our findings demonstrate that multi-strain probiotic supplementation effectively ameliorates both gastrointestinal and neurobehavioral abnormalities in FA mice, potentially through gut microbiota modulation and amino acid metabolic regulation. While we have established the crucial role of the microbiota-gut-brain axis at the microbial level, the causal relationships and detailed mechanisms require further investigation. Additionally, probiotics could exert a beneficial impact beyond metabolites or cytokines, like vagus nerve-dependent manners. Altogether, these data underlined a positive effect of multi-strain probiotics on gastrointestinal disorders and of psychological pathologies, though further preclinical or clinical studies are needed to verify and extend this application.

## Data Availability

The datasets from the current study are available from the corresponding author upon request. The datasets are not publicly available due to being a part of an ongoing study.

## Supplementary Materials

The following supporting information can be downloaded at https://www.mdpi.com/article/10.3390/nu17121955/s1, Supplementary Methods; Table S1: Anaphylaxis scoring scales; Table S2: FDR-Correction for multiple comparisons; Figure S1: Morphological analysis on microglia in PFC; Figure S2: The microbiota-gut-brain axis regulated food allergy induced neurobehavioral changes. References [73,74,75,76] are cited in Supplementary Materials.

## Abbreviations

The following abbreviations are used in this manuscript:

FA	Food Allergy
OVA	Ovalbumin
FMT	Fecal microbiota transplantation
MGBA	Microbiota-gut-brain axis
OFT	Open field test
EPM	Elevated plus test
FST	Forced swimming test
MBT	Marbles berried test
TST	Three-chamber social test
NNAT	Non-selective, non-sustained visual attention test
SI	Social Index
SNI	Social Novelty Index
