# Supplementary material for: Multi-Strain Probiotics Alleviate Food Allergy-Induced Neurobehavioral Abnormalities by Regulating Gut Microbiota and Metabolites
Source: Nutrients. 2025 Jun 8;17(12):1955. doi: 10.3390/nu17121955 (PMC12196041; doi:10.3390/nu17121955)
Supplement: Supplementary file 1 [file nutrients-17-01955-s001.zip › nutrients-3661011-supplementary.pdf]

Supplementary Methods

1. Behavioral experiments

All behavioral tests including open-field test (OFT), elevated plus maze (EPM), forced swimming test (FST), self-grooming, marble burying test (MBT), three-chamber sociability test (TST), non-selective, non-sustained visual attention test (NNAT), and barnes maze test were conducted during the light phase of the illumination cycle. On the test day, mice were transported to the testing room and left in their home cages for at least 1 hour before the tests. EthoVision XT 15 software (Noldus Information Technology Inc. Netherlands) was applied to analyze every behavioral index.

1.1 OFT

The open field with a dimension of 35 × 35 cm surrounded by 50-cm-high walls was used. Mice were individually placed in the same area of the enclosure outside of the center zone and allowed to freely explore for 10 min. The total time spent in the center zone, average speed, distance traveled, and time immobile were recorded to objectively assess anxiety-like behavior as well as overall activity levels.

1.2 EPM

The apparatus consists of two open arms (30 cm × 6 cm × 0 cm) and two perpendicular closed arms (30 cm × 6 cm × 15 cm) extending from a central platform. The entire maze was elevated approximately 80 cm from the floor. Mice were placed in the central zone and allowed to explore the maze freely for 5 min. The number of entries into each arm and the total time spent in each arm were used to evaluate anxiety level. Reductions in the percent of open

arm exploration (time in the open arm divided by the total time in the maze) were interpreted as increased anxiety.

1.3 FST

Mice were placed into a 4 L cylindrical water bath filled with 3 L of water at 24-26 °C under bright light conditions. The test lasted for 6 min and time spent immobile was measured. Immobility was defined as no movement at all or only minor movements necessary to keep the nose above. Higher degrees of depression are indicated with more time spent in immobile.

1.4 Self-grooming

Mice were habituated to an empty cage for 10 min and then timed for spontaneous self-grooming behavior for additional 20 min. Grooming was defined as time spent licking paws, washing the nose and face, or scratching fur with any foot. Grooming time was recorded with a stopwatch by two examiners who were blind to the treatment conditions. Increased cumulative time spent in grooming was interpreted as high level of repetitive behaviors[1].

1.5 MBT

Mice were habituated for 10 min in a clean Plexiglas cage filled with a 5-cm thick layer of sawdust. Following habituation, 20 glass marbles were gently laid out in five rows of four marbles placed equidistantly apart. Mice were then allowed to explore for 10 min. Only marbles covered by 75% or more bedding were counted as buried, and the number of marbles buried was recorded[2].

1.6 TST –

The apparatus was a yellow plexiglass box (60-cm length, 40-cm width) divided into three chambers (20 × 40 cm) by transparency plexiglass walls. A subject mouse was first habituated

to the full empty arena for 10 min, and then entered 2 phases: sociability (stranger 1 vs. empty), and social novelty (stranger 1 vs. stranger 2). In session 1 (sociability), a novel same-sex mouse was placed in one of the small wire cage (Stranger 1), while the other remained empty (Empty). The experimental mouse was allowed to freely explore the three chambers again for 10 min. In session 2 (social novelty), Empty was replaced with Stranger 2, and the test mouse was allowed another 10 min of free exploration in this session. The sides where Empty, Stranger 1, and Stranger 2 were placed were randomly assigned. The time spent exploring each cage was calculated to represented social behaviors.

1.7 NNAT

Modified NNAT was used to test the interest of novel objects exploration and assess the attention levels in mice[3, 4]. Four objects were fixed on to corners of the bottom of the square box. They were all about 1.5 cm in diameter, of the same color (golden) and texture but different in shape. After the 10-min familiarization session, the contact time between the mice and the "new object" was recorded for 10 minutes in test session. The attention level is the percentage of time that they contact with "new objects" divided by total contact time between mice and all four objects.

2. Enzyme-linked immunosorbent assay (ELISA)

Mice were anesthetized with an i.p. injection of pentobarbital and inhalation of isoflurane, sacrificed 2 hours after the allergen challenge. For serum preparation, blood was collected and allowed to coagulate for 2h at room temperature before centrifuged at 3,500×g for 10 min at 4°C. After sacrifice, dissected prefrontal cortex was lysed using a motorized tissue cutter in

lysis buffer (Absin, China, abs9225) and freshly added protease inhibitor cocktail (Thermo Fisher, USA, 1861281). The supernatants were collected after centrifuging at 12000×g for 20min at 4°C, and their protein concentrations were measured using BCA protein assay kit (Beyotime, China, P0010). The serum levels of OVA-IgE (Cayman, USA, 500840), OVA-IgG1 (Chondrex, USA, 3013) and histamine (Bertin, France, A05890) were detected using ELISA kits according to the manufacturer's instructions. The serum and brain concentrations of cytokines were measured using ELISA kits (Abclonal, China) according to the manufacturer's instructions.

3. Immunofluorescence staining and histopathological assessment

Mice for immunofluorescence were subjected to whole body perfusion via a right heart catheter with PBS and then with 4% paraformaldehyde. The whole brains were dissected out from cranial cavity and coronal slices were prepared by Leica CM1950 cryostat microtome. Prefrontal cortex slices were selected according to a brain atlas and incubated in PBS containing 10% normal goat serum (Absin, China, abs933) and 0.1% Triton X-100 (Sigma-Aldrich, USA, T8787) for 2h at room temperature. They were then treated with primary antibodies Iba-1 (1:500 dilution; Wako, Japan, 019-19741) overnight at 4°C in PBS and washed with PBS. The signals were visualized using the Alexa Fluor 594-conjugated secondary antibodies (1:500; Thermo Fisher, USA, A150080). To evaluate neuron and intestine damage, hematoxylin and eosin (HE) (Solarbio Science and Technology, China) and Nissl staining (Solarbio Science and Technology, China) were carried out according to the manufacturer's instructions. The image of the immunofluorescence was captured using a Leica confocal microscope.

4. Fecal microbiome analysis

DNA was purified from fecal specimens using a ZymoBIOMICS DNA kit (Zymo Research, Irvine, CA, U.S.A.). The 16S ribosomal DNA (V3-V4 region) of the microbial DNA extracted from stool sample was amplified with primers (343F 5′-TACGGRAGGCAGCAG515-3′; 798R 5′AGGGTATCTAATCCT806-3′). After PCR, the agarose gel containing target DNA fragments was cut for purification using the DNA Gel/PCR Purification Miniprep Kit (BW-DC3511, Beiwo Meditech Co., Ltd, China) according to the manufacturer's instructions. Taxonomic classification was performed using the plugin q2-feature-classifier64, a taxonomic classifier plugin for the QIIME 2 microbiome analysis platform (https://qiime2.org/), which makes similar calculations using a scikit-learn naive Bayes classifier. Finally, taxonomy was assigned to filtered amplicon sequence variants (ASVs) using a pretrained QIIME2-compatible SILVA version 132 database, with 99% identity for the bacteria and representative sequences.

5. Metabolome analysis

The serum metabolites were analyzed using LC-MS/MS&GC-MS/MS by Shanghai Luming biological technology co., LTD (Shanghai, China). UPLC-Q-TOF/MS (ACQUITY UPLC I-Class, Waters, Massachusetts, USA) and ESI-QTOF/MS (QE plus, Thermo Fisher Scientific, Waltham, MA, USA) were used. The LC-MS/MS analysis was performed by ACQUITY UPLC I-Class plus (Waters Corporation, Milford, USA) fitted with Q-Exactive plus mass spectrometer (Thermo Fisher Scientific, Waltham, MA, USA), and GC-MS/MS was performed by Agilent 7890B gas chromatography system coupled to an Agilent 5977B MSD system (Agilent

Technologies Inc., CA, USA). A two-tailed Student's t-test was further used to verify whether the metabolites of difference between groups were significant. Differential metabolites were selected with VIP values greater than 1.0 and p-values <0.05. The enriched pathway analysis of changed metabolites was performed using KEGG database (http://www.genome.jp/KEGG/pathway.html).

6. Statistical analysis

All data are presented as the mean ± SEM. The statistical significance of the difference between values was determined by t test. A p value < 0.05 was considered statistically significant. All statistical analyses were performed using Graph Pad Prism 7.0 software (Graph Pad, La Jolla, CA, USA).

1. Kalueff, A.V.;Stewart, A.M.;Song, C.;Berridge, K.C.;Graybiel, A.M.Fentress, J.C., *Neurobiology of rodent self-grooming and its value for translational neuroscience.* Nat Rev Neurosci, 2016, **17**, 45-59, DOI: 10.1038/nrn.2015.8.
2. Schwartzer, J.J.;Careaga, M.;Chang, C.;Onore, C.E.Ashwood, P., *Allergic fetal priming leads to developmental, behavioral and neurobiological changes in mice.* Transl Psychiatry, 2015, **5**, e543, DOI: 10.1038/tp.2015.40.
3. Wang, X.;Jiao, X.;Xu, M.;Wang, B.;Li, J.;Yang, F.;Zhang, L.;Xu, L.Yu, X., *Effects of circulating vitamin D concentrations on emotion, behavior and attention: A cross-sectional study in preschool children with follow-up behavior experiments in juvenile mice.* J Affect Disord, 2020, **275**, 290-298, DOI: 10.1016/j.jad.2020.06.043.
4. Millecamps, M.;Etienne, M.;Jourdan, D.;Eschalier, A.Ardid, D., *Decrease in non-selective, non-sustained attention induced by a chronic visceral inflammatory state as a new pain evaluation in rats.* Pain, 2004, **109**, 214-224, DOI: 10.1016/j.pain.2003.12.028.

Supplementary table 1. Anaphylaxis scoring scales.

0	No reaction/clinical symptoms
1	Scratching and rubbing around the nose and head
2	Puffiness around the eyes and mouth, pilar erecti, reduced activity
3	Wheezing, labored respiration and cyanosis around the mouth and tail
4	No activity after prodding or tremor and convulsion
5	Death

Supplementary table 2. FDR-Correction for multiple comparisons.

Taxa (Family level)	FDR-Correction (q) for CON vs. OVA	FDR-Correction (q) for OVA vs. OVA+P
Bifidobacteriaceae	0.098	0.085
Lactobacillaceae	0.232	0.086
Lachnospiraceae	0.269	0.140
[Eubacterium]_coprostanoligenes_group	0.131	0.070
Bacteroidaceae	0.733	0.139
Prevotellaceae	0.232	0.222
Ruminococcaceae	0.213	0.085
Tannerellaceae	0.098	0.048

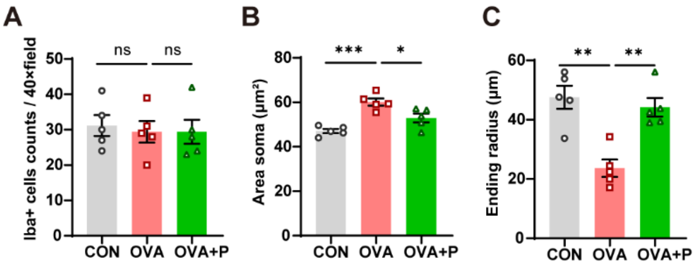

Supplementary Figure 1. Morphological analysis on microglia in PFC

(A) Similar number of Iba+cells in PFC of 3 groups.

(B and C) Microglia of OVA group showed (B) increased area soma and (C) decreased ending radius compared to control ones, while probiotics treatment attenuated these morphological changes.

N=5 in each group. Data are presented as the means ± SEM. ns = not significant. *$p < 0.05$, **$p < 0.01$ and ***$p < 0.001$ using Mann–Whitney U test.

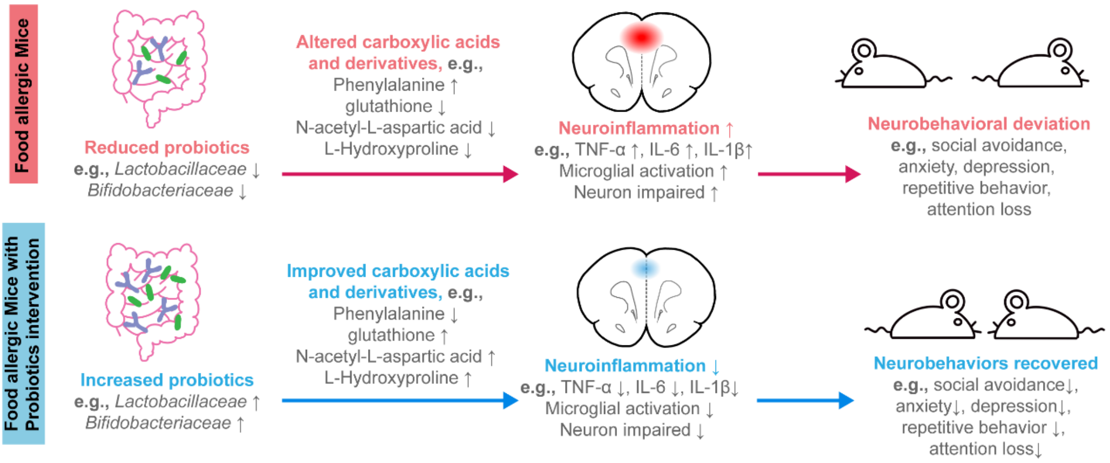

Supplementary Figure 2. The microbiota-gut-brain axis regulated food allergy induced neurobehavioral changes.
